# The effect of Korean pine nut oil (PinnoThin™) on food intake, feeding behaviour and appetite: A double-blind placebo-controlled trial

**DOI:** 10.1186/1476-511X-7-6

**Published:** 2008-02-28

**Authors:** Georgina M Hughes, Emma J Boyland, Nicola J Williams, Louise Mennen, Corey Scott, Tim C Kirkham, Joanne A Harrold, Hiskias G Keizer, Jason CG Halford

**Affiliations:** 1 School of Psychology, University of Liverpool, Eleanor Rathbone Building, Bedford Street South, Liverpool, L69 7ZA, UK; 2 Mennen Training and Consultancy – Junoplantsoen 127, 2024 RP Haarlem, The Netherlands; 3 Lipid Nutrition B V, PO Box 4, 1520 AA Wormerveer, The Netherlands

## Abstract

Certain free fatty acids have been shown to have potent effects on food intake and self-reported changes in appetite; effects associated with increases in the release of endogenous cholecystokinin (CCK) and glucagon like peptide-1 (GLP-1). In the current study, the effects of a Korean pine nut oil product, PinnoThin™, at doses 2 g, 4 g and 6 g triglyceride (TG) and 2 g free fatty acid (FFA), on food intake and appetite were examined in a cross-over double-blind placebo-controlled randomised counter-balanced design in 42 overweight female volunteers. 2 g FFA PinnoThin™, given 30 minutes prior to an ad-libitum buffet test lunch, significantly reduced food intake (gram) by 9% (F(4,164) = 2.637, p = 0.036) compared to olive oil control. No significant effect of PinnoThin™ on macronutrient intake or ratings of appetite were observed. Given the recent data showing that the TG form of PinnoThin™ may also reduce appetite by increasing CCK release, the lack of any effect of the TG form found in this study could be attributed to the timing of the dosing regime. Collectively, these data suggest that PinnoThin™ may exert satiating effects consistent with its known action on CCK and GLP-1 release, and previously observed effects on self-reported appetite ratings.

## Background

Estimations of energy balance suggest that an increase in daily caloric intake of 50 kcal more than energy expenditure can result in a yearly increase in body weight of 1 or 2 kilos [1] contributing to the development of obesity. To correct this positive energy balance one strategy in the arena of weight management and prevention of obesity is the control of food intake via natural appetite suppressants.

The roles of different macronutrients in appetite suppression have been extensively studied. While it has generally been accepted that protein and fibre-rich foods produce strong satiety responses [2,3], high fat foods in particular have been regarded as having a comparatively weaker impact on appetite regulatory mechanisms [4]. In fact a weakened satiety response to fatty meals has been associated with a susceptibility to weight gain [5].

Despite this, dietary fat in the gastro-intestinal tract has been demonstrated to trigger the release of the satiety gut hormones cholecystokinin (CCK) in the proximal small intestine (duodenum), and glucagon like peptide-1 (GLP-1) in the distal small intestine (ileum). The effects of fat on appetite expression have long been associated with the CCK response [6-11]. Moreover, in human volunteers, exogenous CCK infusions reduce food intake and enhance satiety [12-15]. GLP-1 is released in response to nutrients [16,17] such as carbohydrate [18-20] and fat [21,22]. In humans, peripheral GLP-1 infusions have been shown to significantly enhance the satiating potential of a fixed caloric load [23] and also produce a reduction in intake at ad-libitum meals, effects which are associated with reductions in hunger and enhancements in satiety [23-28].

The release of endogenous CCK and GLP-1 suggests, paradoxically, that fat could exert strong effects on the development of satiation and the strength of post-meal satiety, despite an association between high fat over-consumption and obesity [29]. Chain length and degree of saturation may be important properties when considering the effect of fat on the expression of human appetite. Feinle and colleagues [30] demonstrated that duodenal infusions of long chain triglycerides produced greater effects on hunger and plasma CCK levels than medium chain triglycerides, an effect dependent on hydrolysis of the triglyceride (TG) to free fatty acid (FFA) form. Feltrin et al., (2004) [31] also demonstrated that duodenal infusions of FFA of a chain length of C12 produced more potent effects on food intake, appetite expression, CCK and GLP-1 release compared with a FFA of chain length of C10. With regard to saturation, Lawton et al., (2000) [32] demonstrated that polyunsaturated fatty acids (PUFA) of a chain length C18, exert a stronger effect on appetite expression than monounsaturated (MUFA) and saturated (SFA) equivalent C18 forms. Degree of saturation (MUFA versus SFA) also appears to affect endogenous GLP-1 response to high fat meals [33,34]. Therefore, the divergent effects of fats, both in chain length and saturation, on CCK and GLP-1 release, and the expression of appetite, have implications for energy balance [4].

PinnoThin™ is a natural oil pressed from Korean pine nuts (*Pinus Koraiensis*) and consists of 92% of PUFAs and MUFAs, mainly pinolenic acid (C18:3), linoleic acid (C18:2) and oleic acid (C18:1) [35]. Nut consumption is popular worldwide and has been linked with satiety [36,37]. Pine nuts have long been constituent parts of the diets of many cultures, particularly in the Mediterranean and Asian regions, and they are now also consumed very widely outside these geographical areas. In vitro studies on Korean pine nut fatty acids have shown an increase in the release of CCK-8 from STC-1 cells vs. Fatty acids from Italian stone pine nuts and also several other dietary mono and poly unsaturated fatty acids [38]. It has recently been shown that PinnoThin™ stimulates endogenous CCK and GLP-1 release in overweight women when simultaneously consumed with a fixed load breakfast [38,39] and thereby has the potential to reduce prospective food intake.

There is however no information on whether PinnoThin™ can actually reduce food consumption. Therefore, the current study was designed to assess the impact of PinnoThin™ on human food intake and appetite expression. The present study had three aims: 1) to determine whether PinnoThin™, given prior to consumption of a test meal, would produce a significant reduction in kilocalorie (kcal) intake in a subsequent ad-libitum meal, 2) to determine if any hypophagia produced by effective doses of PinnoThin™ were accompanied by appropriate changes in pre- and post-meal appetite ratings indicating any satiety enhancing properties, and 3) to demonstrate that any supplement-induced reductions in food intake were associated with a durable rather than a transient satiety response, consistent with the reported physiological mechanism of action. By examining ad-libitum intake at an evening meal it will be possible to determine if any initial reductions in food intake produced by PinnoThin™ are compensated for by over consumption at later eating opportunities.

## Results

### Participants

**Figure 1:**
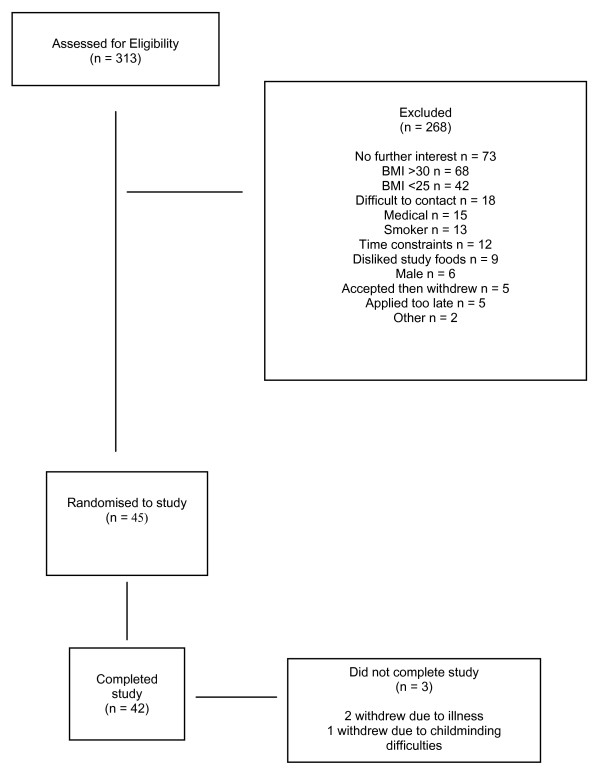
Total enquiries, participants screened and recruited to study.

The study commenced in June 2006 and was completed in February 2007. In total, 45 women were enrolled onto the study. Of the 45 enrolled, 42 participants completed all five conditions. One participant completed one morning and lunch session of one visit and withdrew due to unrelated illness; one participant completed one full visit but withdrew from the study due to illness, the cause of which was unascertained; and one participant completed two visits before withdrawing due to childminding problems. Figure 1 shows details of the number of enquiries, participants screened and outcome. The demographic (age), and anthropometric (weight, height, BMI) characteristics of the completing participants, together with the screening measure scores are shown in Table 1.

**Table 1:** Baseline characteristics of subjects who completed the study (mean ± SD)

	**Mean (N = 42)**
Age (years)	33.8 ± 15.6
Weight (kg)	73.9. ± 7.0
Height (m)	1.7 ± 0.7
BMI (kg/m2)	27.4 ± 1.3
DEBQ-R	3.0 ± 0.7
CES-D	11.3 ± 7.9

### Intake at test meals

Intake at lunch and supper together with combined intake at lunch and supper (in grams and kcal) are shown in Table 2. There was a significant main effect of condition on food intake at the test meal (F(4,164) = 2.637, p = 0.036). A decrease in both the amount and energy content of the food consumed at the buffet meal was seen in the PinnoThin™FFA condition. This dose reduced gram intake by 9% with post-hoc tests showing a significant difference between the 2 g PinnoThin™FFA and placebo conditions (t (41) = 2.270, p = 0.029). With regard to effect of condition on energy intake an insignificant trend was seen (F(4,164) = 1.552, p = 0.190). Energy intake was reduced by 7% from control in the PinnoThin FFA condition with post-hoc tests showing a near significant effect (t(41) = 1.702, p = 0.096). The results stayed the same after exclusion of outliers. No effects were seen for the other conditions and no differences were seen in intake during supper. No main effect of condition was found for macronutrient content or the type of food.

**Table 2:** Macronutrient, energy and gram intakes at the test meals (mean ± SE)

Test Meal Intake	Placebo	PThin TG 2 g	PThin TG 4 g	PThin TG 6 g	PThin FFA 2 g
**LUNCH**					
Protein (g)	24.4 ± 1.5	24.5 ± 1.5	23.7 ± 1.5	24.6 ± 1.4	22.3 ± 1.4
Fat (g)	32.0 ± 2.1	32.1 ± 2.3	30.1 ± 1.9	32.4 ± 1.9	29.1 ± 2.0
Carbohydrate (g)	79.8 ± 5.4	82.3 ± 5.4	85.6 ± 5.6	81.7 ± 5.7	75.9 ± 5.0
Gram Intake (g)	380.2 ± 18.5	373.7 ± 21.3	386 ± 18.8	383.7 ± 18.2	349.7 ± 18.8*
Energy Intake (kcal)	706.1 ± 38.9	717.7 ± 41.6	709.5 ± 37.5	718.8 ± 38	656.2 ± 36.8
					
**SUPPER**					
Protein (g)	37.9 ± 2.0	35.6 ± 2.0	36.7 ± 1.9	36.1 ± 1.7	37.4 ± 1.9
Fat (g)	21.0 ± 1.1	20.6 ± 1.1	20.6 ± 0.9	20.9 ± 1.2	21.6 ± 1.0
Carbohydrate (g)	165.6 ± 9.4	159.6 ± 8.8	166.2 ± 8.8	161.9 ± 8.0	170.3 ± 8.4
Gram Intake (g)	521.4 ± 27.4	508.4 ± 29.7	523.4 ± 25.9	513.7 ± 25.2	530.2 ± 24.1
Energy Intake (kcal)	1002.4 ± 50.8	965.4 ± 49.8	996.1 ± 46.3	979.1 ± 44.5	1024.4 ± 46.2
					
**LUNCH & SUPPER**					
Protein (g)	62.4 ± 2.7	60.1 ± 2.9	60.4 ± 2.6	60.7 ± 2.4	59.7 ± 2.7
Fat (g)	53.0 ± 2.4	52.7 ± 2.7	50.6 ± 2.2	53.3 ± 2.2	50.8 ± 2.5
Carbohydrate (g)	245.4 ± 9.5	241.9 ± 10.8	251.8 ± 9.5	243.6 ± 9.6	246.2 ± 9.6
Gram Intake (g)	901.6 ± 39.3	882.1 ± 45.9	909.4 ± 38.3	897.4 ± 37.1	879.8 ± 38.5
Energy Intake (kcal)	1708.5 ± 59.6	1683.1 ± 69.1	1705.6 ± 55.6	1698 ± 58.9	1680.7 ± 62.5

* significantly different from placebo *p *< 0.05

### VAS ratings of appetite

There were no significant effects of condition on subjective ratings of hunger, fullness, satisfaction, desire to eat, prospective consumption, thirst or nausea. Additional ANOVAs to investigate trends were run from the pre-dose VAS ratings (T6) through to the post supper rating (T13). No statistically significant scores were found.

### Adverse events

No signs or symptoms of serious adverse events due to the study product were reported. One participant reported blotchiness on the palms of her hands which occurred four days after the last visit. Un-blinding of the product revealed the final visit was placebo and therefore the event was completely unrelated to the product.

## Discussion

The use of natural appetite suppressants may be a key strategy to help prevent weight gain. Nuts have been studied for their satiation ability and contain fiber, protein and oil and are highly nutritious. Many cultures use Korean pine nuts in popular dishes. Korean Pine nut oil (PinnoThin ™) which is high in pinolenic acid has previously been shown to increase CCK in STC-1 cell culture and to have effects on satiety in response to a fixed caloric load, such as increasing concentrations of CCK and GLP-1 in humans [38]. Specific changes in satiety measures was observed in prospective food consumption [38,39]. The purpose of the study was to evaluate the effects of PinnoThin™ on subsequent food intake in an ad-libitum meal. PinnoThin™FFA produced a significant 9% reduction in ad-libitum food intake thirty minutes after dosing compared with the placebo control. No differences between PinnoThin™ and the placebo were seen at the evening meal, suggesting that there is no compensation for the lesser food intake during lunch. No effect was produced by any of the three doses (2 g, 4 g or 6 g) of the PinnoThin™ TG.

No adverse effects related to the study product were reported by participants during the full study period or at post-study debriefing. No significant effect of product on nausea was observed on study days. This indicates that PinnoThin TG at various doses and PinnoThin FFA 2 g were all well tolerated by the forty-two participants who completed the study.

The reduced food intake of 9% corresponded to a 7% (50 kcal) reduction in energy intake. Although this reduction was not statistically significant, probably due to lack of power (power calculations were based on a 10% difference), a reduction of 50 kcal is potentially biologically relevant. Brown et al [1] estimated that the average weight gain in a female population equates with an energy imbalance of only 10–40 kcal per day. Post-hoc paired t-test revealed that the difference in kcal intake between the control and FFA condition would have been significant in a one-way test. The slight disparity between the magnitude of gram and kcal intake reduction between the control and the FFA condition could suggest small treatment induced effects of food choice i.e. a greater reduction in the intake of less energy dense foods. However, no difference was seen between the intake of macronutrients, sweet/savoury or high/low fat foods.

Both the PinnoThin™ FFA and TG forms have been shown to have an effect on satiety by increasing levels of CCK [38]. While the PinnoThin FFA reduced food intake, the lack of effect of any dose of the PinnoThin™TG on food intake in this trial remains unclear. Feinle et al., (2001) [30] demonstrated that duodenal infusions of a long chain TG can produce potent effects on plasma CCK levels and significantly reduce hunger. This is consistent with the known mechanisms of CCK release [11,40]. In the Feinle study, effects of TG on CCK response and appetite were blocked by the lipase inhibitor tetrahydrolipstatin. Therefore, the uptake and metabolism of the precursor triglycerides to the FFA form, the latter of which is responsible for CCK and GLP-1 release, is needed to induce satiety.

In the current study, the digestion of pine nut oil TG to FFA form was not assessed. However, there is no reason to believe that the actions of lipases were inhibited in any way. The lack of effect may be explained by insufficient time passed between the intake of the TG capsules and the ad-libitum lunch for lipase action to convert sufficient TG to FFA. In this way there could have been an insufficient rise in endogenous CCK response to significantly reduce intake at any TG dose. In support of this notion, it is known that uptake of fat from capsules taken with water and without simultaneous ingestion of food is delayed (personal communication from Dr Rob Havenaar TNO Research Zeist, The Netherlands). Also, in a previous study with PinnoThin™ the effects on CCK response took longer to develop for the TG compared to the FFA (60 min rather than 30 min) [38]. A long term trial including 188 overweight men and women showed that pinolenic acid was present in the blood at 4 and 8 months after daily supplementation with 3 g of PinnoThin™ TG whereas none was present in the control (olive oil) group (unpublished results). This shows that PinnoThin™ TG is normally taken up and digested, i.e. FFA are released after ingestion of PinnoThin™TG. When comparing FFA to TG it also has to be taken into account that the total amount of FFA will become available, whereas only two out of the three fatty acids will become available for the TG. An effect seen after 2 g of FFA should therefore be compared with an effect of at least 3 g for the TG.

The mechanism of effect of PinnoThin™ is based on the increase in satiety hormones seen in an earlier study [38]. The effect of PinnoThin™ on intestinal hormone release is most likely mediated through chylomicron formation. Fatty acids with a chain length of more than 12 carbon atoms are absorbed into the circulation as chylomicrons. A study in rats has shown that suppression of food intake by long chain fatty acids is inhibited by blocking of chylomicron transport through specific inhibitors [41]. CCK signaling pathways are closely related to the transport of chylomicrons [42]. The fact that only fatty acids with more than 12 carbon atoms induce CCK speaks in favour of this mechanism. This suggests that the fatty acids from PinnoThin™ particularly affect chylomicron formation or transport and thereby influence release of CCK. This release leads to a delay in gastric emptying and in turn to an increased feeling of satiety and a decreased appetite. Overall, despite the lack of the TG form to affect intake or appetite in this trial, these data are consistent with the notion that fats have divergent effects on the expression of appetite.

However, whilst CCK release from the proximal small intestine may be critical in the acute effects of PinnoThin™ on within meal satiation or immediate post meal satiety, such effects may not explain any observed reductions in food intake across the whole day or any purported long-term effects body weight. A variety of CCK anti-obesity drugs in early clinical development have failed to progress in later clinical trials presumably in part because of a lack of efficacy beyond an initial reduction in hunger. PinnoThin™ induced GLP-1 released from the distal small intestine may be of more importance in suppressing post-meal intake by sustaining inter-meal satiety. The relative contributions of these two hormones to the effects of PinnoThin™ on within-meal satiation, post-meal satiety and associated changes in body weight over repeated dosing remain to be clarified. In should also be noted it has been shown that co-administration of CCK (in the form of CCK-33) and GLP-1 does not produce an additive suppression of food intake, although their effects on pre-meal hunger were greater than when administered separately [43].

## Conclusion

PinnoThin™ in both FFA and TG forms was well tolerated. PinnoThin™ FFA employed in this study produced a reduction in gram food intake consistent with previously reported effects on appetite and CCK response. The lack of significant effects of PinnoThin™TG on food intake and appetite does not necessarily suggest that the TG form is ineffective. Comparative data on PinnoThin™ FFA and TG forms has become available since the study design which suggests that the TG produces a delayed response on gut hormone release. The time taken for TG to break down into FFA form in the human gut is unknown, and this could explain the delay. Similarly the effects of TG on gut hormone release appear to differ in magnitude.

## Materials and methods

### Participants

Forty-two healthy women, aged 18–64, with a body mass index (BMI) between 25–30 kg/m^2 ^completed the study. Volunteers were recruited by advertisement from the University of Liverpool and surrounding area of Merseyside in the North West of England. Upon response to the advertisements, individuals completed a standardized telephone or email assessment to initially determine their eligibility for the study. The specific inclusion criteria were age between 18 and 65, overweight with a BMI of 25–30 kg/m^2^, non-smoker, interested in weight loss but not currently dieting, and able to attend the study centre at the requisite times to eat the foods offered.

### Screening

After the initial telephone/email assessment, potential participants received detailed information on the protocol, completed a medical history and were invited to the study centre, (The Kissileff Ingestive Behaviour Laboratory in the School of Psychology, The University of Liverpool), for a screening no more than 21 days before commencing the study. All volunteers signed an informed consent form before any study-specific procedures were undertaken. The consent form stated that participants had read and understood the information sheet. The protocol and consent form were approved by the School of Psychology Research Ethics Committee on May 15^th ^2006. The study conformed to the British Psychological Society Code of Practice and the UK Economic and Social Research Council (ESRC) Research Ethics Framework (REF), and was also in line with the relevant sections of the Declaration of Helsinki. Volunteers received financial compensation for their participation.

At the full screening, height, using a stadiometer to the nearest cm, and weight, using standard calibrated scales to the nearest 0.1 kg, were verified. Participants also completed a diet history, the restraint sub-scale of an eating behaviour questionnaire [The Dutch Eating Behaviour Questionnaire (DEBQ-R)] [44], and a depression scale [Center for Epidemiologic Studies Depression Scale (CES-D)] [45].

### Exclusion criteria

Following screening, participants were excluded from the study if they reported any of the following: significant health issues likely to affect their well-being and/or appetite; gastrointestinal symptoms requiring treatment; taking medication known to affect appetite; systemic or local treatment likely to interfere with evaluation of the study parameters; bariatric surgery; current adherence to a specific food avoidance diet; pregnancy; planning to become pregnant or breastfeeding; general food allergies or specific allergies to milk, lactose, nuts, olives or any of the study foods; dislike of more than 20% of the ad-libitum study foods; extreme dietary restraint (>2 standard deviations from the DEBQ-R mean) and significant levels of depression (>16 on the CES-D). Women who were employed in nutrition, dietetics, food research or the food manufacturing industry were also excluded from the study.

### Study design

This was a double-blind, placebo-controlled study using a randomized crossover design to evaluate the effects of four weight-loss supplement doses against a placebo control. Study doses were administered acutely in a counterbalanced sequence. Each treatment was separated by a week. At the end of five visits every participant had received each of the five treatments. The sample size was calculated on the basis of the previous clinical trial of the FFA form of PinnoThin™ [38]. Randomisation to the study was by means of Latin squares [46]. Allocation of participants to condition using the Latin Square was performed by the experimenter. The company independently randomly assigned the five doses to the five conditions (A-E).

### Materials and tools

Amount of food and water was recorded by weight scales (Sartorius Model BP8100, Sartorius Ltd., Epsom, UK; 0.1 g accuracy) before and after meals to ascertain intake. Visual Analogue Scales (VAS) were used to rate degrees of hunger, fullness, satisfaction, desire to eat, perception of how much participants could eat (prospective consumption), thirst and nausea. VAS consist of 100 mm horizontal lines anchored by "not at all" and "extremely" at opposite ends, upon which participants record with a vertical line their subjective ratings. For example, hunger was rated along a 100 mm line that was preceded by the question "how hungry do you feel at this moment?" and anchored on the left by "not at all hungry" and on the right by "extremely hungry". These questionnaires were completed immediately before and after each meal, before the supplement and at hourly intervals throughout the test day [47-49].

PinnoThin™ and placebo were supplied in the form of six capsules per dose per test day. Each capsule contained 1 g of oil and in total the subjects received 2 g, 4 g or 6 g PinnoThin™ Triglyceride (TG), or 2 g of PinnoThin™ free fatty acid (FFA), with 2, 4 or 6 g of olive oil (placebo) (Table 3). Olive oil was chosen as a placebo, based on the fatty acid content of PinnoThin™ and its wide use in the diet. Olive oil consists mainly of oleic acid, which is also found in PinnoThin™ (25%). Furthermore, the other main fatty acids in PinnoThin™ are linoleic (46%) and pinolenic acid (15%), both having the same chain length as oleic acid (C18). Olive oil was therefore thought to be an appropriate control. The fatty acid components of the product and placebo are shown in Table 4. The capsules were packaged in coded containers labelled A to E to ensure the double-blind status of the study.

**Table 3:** Content of study six capsules given 30 minutes before lunch in the five experimental conditions

	Capsule 1	Capsule 2	Capsule 3	Capsule 4	Capsule 5	Capsule 6
Placebo	1 g Placebo (olive oil)	1 g Placebo (olive oil)	1 g Placebo (olive oil)	1 g Placebo (olive oil)	1 g Placebo (olive oil)	1 g Placebo (olive oil)
2 g FFA	1 g FFA	1 g FFA	1 g Placebo (olive oil)	1 g Placebo (olive oil)	1 g Placebo (olive oil)	1 g Placebo (olive oil)
2 g TG	1 g TG	1 g TG	1 g Placebo (olive oil)	1 g Placebo (olive oil)	1 g Placebo (olive oil)	1 g Placebo (olive oil)
4 g TG	1 g TG	1 g TG	1 g TG	1 g TG	1 g Placebo (olive oil)	1 g Placebo (olive oil)
6 g TG	1 g TG	1 g TG	1 g TG	1 g TG	1 g TG	1 g TG

**Table 4:** Olive and Korean Pine Nut oils

**Product**	**Fatty Acid Components**		
PinnoThin	15% pinoleic	25% oleic	45% linoleic
Olive oil	12% palmitic	71% oleic	10% linoleic

### Study procedure

On each day preceding the study day participants were asked to keep their food intake, fluid intake and activity levels similar and to record these in a diary from 5 pm until they retired for the night. They were asked not to consume any alcohol and not to eat or drink anything except water from 12 midnight until they attended the study centre the following morning.

At 8.30 am on each test day participants attended the study centre where they consumed a fixed-load breakfast (496 kcal; Table 5). Participants were then free to leave the study centre. They were instructed not to eat or drink anything except water that was provided by the study. Thirty minutes before lunch (three and a half hours after breakfast) the 6 capsules were provided with 200 ml water. They were instructed to swallow all the capsules with as much of the water as required. Following a rest period an ad-libitum lunch was served (Table 6). Participants were instructed to eat as much as they liked from the choice of foods and water offered, taking as long as they wished, and signalling when they had finished. Participants were then free to leave the study centre, with instructions not to eat or drink anything except the provided water. Participants returned for supper four hours later and were served a hot ad-libitum evening meal (Table 6). Again, they were asked to consume as much as they wanted, taking as long as they wished and signalling when they had finished. The study day was then complete and participants were not given any food restrictions for the remainder of the test day. As stated, VAS were completed throughout the day. The same procedure was followed on all test days.

**Table 5:** Amount and energy composition of fixed load breakfast

**Food**	**Amount (g)**
Kellogg's Cornflakes	30
Semi-skimmed UHT Milk	125
Orange Juice	200
Sliced White Bread (toasted)	60
Flora Margarine	10
Strawberry Jam (+ hot drink, 35 g milk and sugar if required)	20

**TOTAL WEIGHT**	**445**
TOTAL KCALS 496	

10% Energy from Protein, 17% Energy from Fat, 73% Energy from Carbohydrate

**Table 6:** Nutritional content of foods offered at test meals

**Food**	**No of Items**	**Amount (g in serving)**	**Protein (g in serving)**	**Fat (g in serving)**	**CHO (g in serving)**	**Kcal in serving**
**LUNCH**						
Tesco Medium Sliced White Bread	4 slices	144	11.66	2.30	65.52	329.76
Kallo Low Fat Rice Cakes	4 pieces	30	2.40	0.84	23.61	111.60
Asda Turkey Slices	5 slices	62.5	11.69	3.13	1.50	80.63
Tesco Danish Salami	5 slices	44.6	5.89	20.52	3.12	220.78
Tesco Value Cottage Cheese	serving	150	21.45	2.25	5.10	127.50
Asda Cheese Substitute	serving	100	25.00	21.10	2.50	300.00
Flora Light Margarine	4 packs	40	Trace	2.36	Trace	21.20
Butter	4 packs	27.2	0.14	22.22	Trace	200.46
Tomato	Approx. 2	135	Trace	Trace	Trace	Trace
Cucumber	serving	80	Trace	Trace	Trace	Trace
Lettuce	Approx. 1/4	100	Trace	Trace	Trace	Trace
Walker's Ready Salted Crisps	1 packet	25	1.63	8.50	12.25	132.50
Tesco Value Jaffa Cakes	4 cakes	45.2	2.17	3.98	30.56	167.24
Tesco Jelly Babies	20 sweets	120	6.36	0.00	96.84	412.80
Tesco Chocolate Mousse	1 pot	67	2.41	5.63	18.49	134.00
Asda Apple Slices	1 pack	80	0.24	0.08	9.60	40.00
Tesco Value Chocolate Chip Cookies	serving	100	4.80	26.10	64.70	513.00
Asda Smart Price Cheese and Tomato Pizza	1	66.5	4.66	5.32	26.60	172.90
Mayonnaise	5 packets	60	0.96	48.60	0.96	415.39
Mustard	serving	36	2.52	3.20	6.01	63.00
						
**SUPPER**						
Tesco Fusilli	serving	250	33.00	5.00	171.25	862.50
Tesco Value Garlic Bread	3 slices	50	4.05	6.55	21.00	159.00
Tesco Value Fruit Cocktail	1 tin	390	1.56	0.39	42.90	183.30
Ragu Pasta Sauce	1 jar	460	5.40	0.45	31.50	148.50
Asda Cheese Substitute	serving	50	12.50	10.55	1.25	150.00
Tesco Dark Choc Ice	1 bar	80	2.48	16.96	21.92	248.00

### Test meals

A standard fixed-load breakfast (496 kcal) was dispensed to participants in all conditions (Table 5). In addition to the fixed-load breakfast, at the first visit, participants were offered a hot drink of tea or coffee with additional milk (35 g) and sugar if desired. If requested, this drink had to be consumed on each subsequent visit. The ad-libitum lunch consisting of a selection of 20 cold items, is shown in Table 6. Supper was an ad-libitum hot pasta meal with a selection of desserts (Table 6). The ad-libitum meals (lunch and supper) were designed to offer a selection of high and low fat savoury and sweet food items. The nutritional content of all food items is also shown in Table 6.

Lunchtime was fixed at precisely 4 hours after breakfast and supper 4 hours after lunch. All meals were served in individual booths in the test study centre.

### Statistical analysis

The primary outcome, intake at the test meals, was analysed for amount consumed (in grams and kcal) using within-subject analysis of variance (ANOVA) with condition (0 g, 2 g, 4 g, 6 g PinnoThin TG, and 2 g PinnoThin FFA) as a within-subject factor. Because breakfast was fixed, this was not included in the statistical analysis. Intake at lunch was analysed in relation to macronutrient content using a 2-way within-subjects ANOVA with condition (0 g, 2 g, 4 g, 6 g PinnoThin TG, and 2 g PinnoThin FFA) and macronutrient (fat, carbohydrate and protein) as within subject factors. The 20 food items offered at the ad-libitum lunch were further analysed according to variation in fat content and taste. Foods were grouped into high fat savoury (HFSA), low fat savoury (LFSA), high fat sweet (HFSW), low fat sweet (LFSW) and fruit and vegetables (FTVG) sets. These food groups were analysed using a 2-way within-subjects ANOVA with condition (0 g, 2 g, 4 g, 6 g PinnoThin TG, and 2 g PinnoThin FFA) and taste (HFSA, LFSA, HFSW and LFSW) as within-subjects factors. The secondary outcome measures of the study were changes in ratings of appetite such as hunger, fullness, prospective consumption, and desire to eat, assessing the nature of any product-induced reductions in food intake. These parameters rated on the VAS were analysed using within-subject ANOVA for repeated measures with condition (0 g, 2 g, 4 g, 6 g PinnoThin TG, 2 g PinnoThin FFA) and time (pre-breakfast, post-breakfast, 10 am, 11 am, 12 pm, pre-dose, pre-lunch, post-lunch, 2 pm, 3 pm, 4 pm, pre-supper and post-supper) as within-subject factors. If a time-by-condition interaction effect was found significant, paired t-tests would have been conducted at each rating time between conditions. Z-score analysis (individual scores measured against the mean of all other scores) was used to identify outliers. Scores of <-2 or >+2 were considered outliers and analysis was re-run following removal of outliers. Analysis was performed using SPSS for Windows, Version 13 (SPSS Inc., Chicago, US). The assumptions of the ANOVA model were tested and if homogeneity of variance was not found, multivariate tests were adopted for that variable. All tests are two-tailed unless stated. The data remained blinded until after the primary and secondary outcomes were analysed.

## Competing interests

Lipid Nutrition is involved in research/development and marketing/sales of Pinnothin as a satiety ingredient. The general goal of Lipid Nutrition is to develop and sell lipids and oils with a scientifically proven health benefit. Pinnothin consists of Korean pine nut oil. Therefore Lipid Nutrition has a commercial interest in this publication. The University of Liverpool, the conducting laboratory, was paid by Lipid Nutrition to perform and report the scientific work which formed the basis of this publication. The University of Liverpool (Jason Halford's laboratory) and Lipid Nutrition declare that the data presented in this publication represent a true and faithful representation of the work performed.

## Authors' contributions

Louise Mennen and Jason C G Halford participated in the study design, in the analyses and interpretation of the data and writing of the manuscript. Georgina M Hughes, Emma J Boyland, Nicola J Williams, Tim C Kirkham and Joanne A Harrold were involved in performing the studies and writing the manuscript. Corey Scott was involved in writing the manuscript. Hiskias G Keizer was involved in writing the manuscript and in the submission of this paper. Jason C G Halford is corresponding author of this study. All authors read and approved the final manuscript.
